# Sedimentation of a starch microsphere: What is usually missed and why?

**DOI:** 10.1016/j.heliyon.2023.e20257

**Published:** 2023-09-21

**Authors:** Ivan Argatov, Nedim Krcic, Vitaly Kocherbitov

**Affiliations:** a Institut für Mechanik, Technische Universität Berlin, 10623 Berlin, Germany; b Magle Chemoswed, Agneslundsvägen 27, SE-212 15, Malmö, Sweden; c Faculty of Health and Society, Malmö University, SE-205 06 Malmö, Sweden; d Biofilms – Research Center for Biointerfaces, Malmö University, SE-205 06 Malmö, Sweden

**Keywords:** Sedimentation, Starch microspheres, Non-laminar flow, Settling time, Settling distance

## Abstract

Gravimetric sedimentation is known as a relatively simple method of determining density of spherical particles. When the method is applied to water-swollen starch microparticles of about submillimeter sizes, it becomes evident that a careful selection of the experimental setup parameters is needed for producing accurate testing results. The main reason for this is that the mean particle density is very close to the density of water, and therefore, a dynamic model accounting for the so-called Bassett history force should be employed for describing the unsteady accelerating particle settling. A main novelty of this study consists in deriving *a priori* estimates for the settling time and distance.

## Graphical abstract

**Figure:**
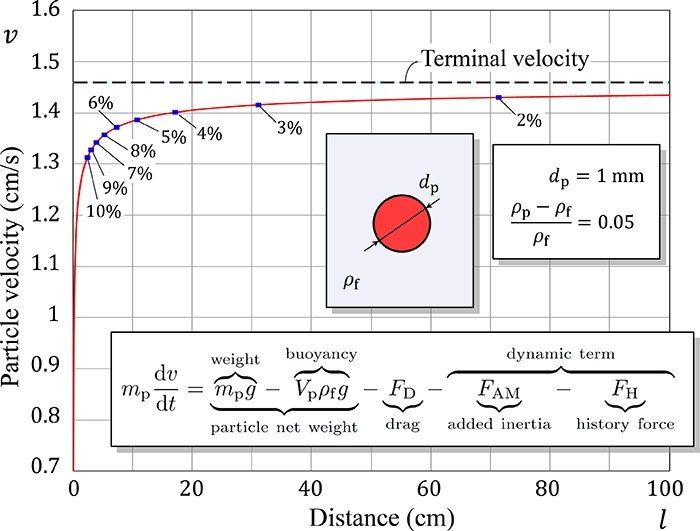

## 1 Introduction

Starch microparticles find diverse applications in pharmacy, e.g., for designing drug delivery systems [1]. In practice, the starch microparticle size (in diameter, $d_{p}$) can be easily produced in the range from a few micrometers to more than one millimeter [2]. Owing to their small size starch microparticles are amenable to sedimentation studies [3]. The method of sedimentation finds multiple applications, including the separation of nanoparticles and cells of differing density [4].

Since starch particles are susceptible to pronounced swelling in water, both the particle volume, $V_{p}$, and the particle mass, $m_{p}$, can vary considerably in transition from dry to wet conditions. The knowledge of both the particle mass and the particle volume is required in the evaluation of the mean particle density ${\overset{¯}{\rho }}_{p}=m_{p}/V_{p}$. Using the method of sedimentation analysis [5] for a single particle, it is possible to estimate the density difference $\Delta \rho ={\overset{¯}{\rho }}_{p}-\rho_{f}$, where $\rho_{f}$ is the density of fluid.

Usually, determination of the mean particle density ${\overset{¯}{\rho }}_{p}=\rho_{f}+\Delta \rho$ from gravimetric sedimentation is based on Stokes' law, which yields

(1) $$\Delta \rho =\frac{18\mu_{f}}{gd_{p}^{2}}v_{s},$$

where *g* is the gravitational acceleration, $\mu_{f}$ is the fluid viscosity, and $v_{s}$ is the steady-state (or terminal velocity) of the sedimenting particle, which is routinely assessed in experiment [5].

It is well known that Stokes' analytical solution for the drag force acting on a rigid sphere moving in an unbounded fluid medium applies to Newtonian fluids in the laminar flow regime, which is characterized by low values (at least less than unit [6]) of the particle Reynolds number

(2) Res=ρfdpμfvs.

Under the assumption that Res≤0.5, Eq. (1) and (2) imply the following upper limit for the particle diameter to ensure particle settling in the Stokesian regime [7]:

(3) $$d_{p}\leq \sqrt[{3}]{\frac{9\mu_{f}^{2}}{g\rho_{f}\Delta \rho }}.$$

Thus, in view of the upper bound inequality (3), the application of Stokes' law in sedimentation studies is practically limited to relatively small particles. In regard to starch microparticles, it is known that the bulk dry density of Starch varies in the range 1.25–$1.50\;g/{\mathrm{cm}}^{3}$ depending on the Starch type and treatment [8]. Moreover, with increasing moisture content, the volumetric swelling ratio can reach values from 5 to 7 (in exceptional cases it can be higher up to 15–25), thereby significantly lowering the mean particle density [9]. For instance, estimating the particle difference Δ*ρ* to be equal to $0.02\;g/{\mathrm{cm}}^{3}$ for water-swollen starch microspheres and taking the characteristic values $g=9.87\;m/s^{2}$, $\rho_{f}=0.997\;g/{\mathrm{cm}}^{3}$, and $\mu_{f}=0.89\cdot 10^{-3}\;\mathrm{Pa}\cdot s$, we readily find that the Stokes law-based formula (1) is applicable to starch microspheres in diameter less than 320 μm. That is why, the application of the method of sedimentation for starch microparticles of larger size [10] requires another approach which accounts for finite values of Reynolds number.

A critically important issue is to not only acknowledge the non-linear dependence of the drag force on the particle velocity but also to recognize that a certain time interval is required for a particle to come close to the terminal velocity. Namely, based on the previous calculations of [11], Bernhardt [12] has presented the following estimate

(4) $$t_{99}\approx 800\frac{\rho_{f}d_{p}^{2}}{\mu_{f}}$$

for the time $t_{99}$ for which the particle velocity equals $0.99v_{s}$.

Observe that *a priori* estimates for characteristic time and distance values in the Stokesian regime were given in [7], which however, sufficiently differ from the approximate formula (4) for large solid particles in liquids. The reason for this lies in the employed equation for describing non-stationary movement of a sedimenting particle. Indeed, according to Newton's second law, the dynamic balance between the inertial force acting on the particle and external forces can be represented as

(5) $$m_{p}\frac{dv}{dt}=\underset{\mathrm{particle net weight}}{\underset{︸}{\overset{\mathrm{weight}}{\overset{︷}{m_{p}g}}-\overset{\mathrm{buoyancy}}{\overset{︷}{V_{p}\rho_{f}g}}}}-\underset{\mathrm{drag}}{\underset{︸}{F_{D}}}-\overset{\mathrm{dynamic term}}{\overset{︷}{\underset{\mathrm{added inertia}}{\underset{︸}{F_{\mathrm{AM}}}}-\underset{\mathrm{history force}}{\underset{︸}{F_{H}}}}},$$

where $F_{D}$ is the drag force, $F_{\mathrm{AM}}$ is the added mass force, and $F_{H}$ denotes the so-called Basset history force, which depends on the history of the particle acceleration and is completely neglected in the model of particle settling in laminar flow conditions [7].

So, in the steady state, when $v\equiv v_{s}$, both the inertial force and the dynamic term in Eq. (5) vanish, and the force balance simplifies as

(6) $$F_{D}=(m_{p}-V_{p}\rho_{f})g.$$

In the Stokesian regime, $F_{D}=3\pi \mu_{f}d_{p}v$, and Eq. (6) yields formula (1). In general case, Eq. (6) serves as a basis for determining the terminal velocity $v_{s}$.

What remains missing is an *a priori* estimate for the distance that the particle travels while approaching the steady state. To the best of the authors' knowledge there are no simple solutions for the characteristic distance based on the dynamic equation including the Basset history term, and the aim of the present study is to fill this gap.

## 2 Dynamic model of sedimentation

### 2.1 Dynamic force balance

The vertical motion of a spherical particle in a stationary medium under the action of gravity is governed by Newton's second law

(7) $$m_{p}\frac{dv}{dt}=(m_{p}-V_{p}\rho_{f})g-F_{D}-F_{\mathrm{AM}}-F_{H},$$

where $m_{p}$ is the particle mass, $\rho_{f}$ is the particle velocity, *t* is the time variable, the derivative dv/dt represent the particle acceleration, $V_{p}$ denotes the particle volume, $\rho_{f}$ is the fluid density, *g* is the gravitational acceleration, $F_{D}$ is the quasi-steady-state drag force, $F_{\mathrm{AM}}$ is the so-called added (or virtual) mass force, and $F_{H}$ is the unsteady-drag (or “history”) force.

It is to note that in contrast to a more general dynamic model of motion for particles developed in [13], Eq. (7) neglects forces that may be generated by rotation of the particle as well as by the fluid shear and the fluid stress gradient. In experiments on sedimentation of a single spherical particle in a stationary fluid medium, the latter forces are negligible.

For a spherical particle, we have

(8) $$V_{p}=\frac{\pi }{6}d_{p}^{3},\;m_{p}=V_{p}{\overset{¯}{\rho }}_{p},$$

where $d_{p}$ is the particle diameter, and ${\overset{¯}{\rho }}_{p}$ is the mean particle density.

The quasi-steady drag force is defined as

(9) $$F_{D}=\frac{1}{2}\rho_{f}v^{2}A_{p}C_{D},$$

where $A_{p}$ is the projected area of the particle, and $C_{D}$ is the drag coefficient, which, generally speaking, depends on the particle velocity *v*.

For a spherical particle, we have

(10) $$A_{p}=\frac{\pi }{4}d_{p}^{2}.$$

The added mass force, which is caused by the inertia of the fluid surrounding the particle, can be evaluated as follows:

(11) $$F_{\mathrm{AM}}=\frac{1}{2}\rho_{f}V_{p}\frac{dv}{dt}.$$

The so-called history force accounts for the unsteady component of the surface force due to the temporal development of the viscous region surrounding the particle and can be approximated using the window model developed by Dorgan and Loth [14] as follows:

(12) $$F_{H}=\frac{3}{2}d_{p}^{2}\sqrt{\pi \rho_{f}\mu_{f}}\int_{t-t_{\mathrm{window}}}^{t}\frac{dv(t^{\prime })}{dt^{\prime }}\frac{dt^{\prime }}{\sqrt{t-t^{\prime }}}.$$

Here, $\mu_{f}$ is the dynamic viscosity of the fluid, $t^{\prime }$ is the integration variable, and the lower limit of integration is given by

t−twindow=min⁡{0,t−tH},tH=ρfdp2μf(0.502Re+0.123)2,

with $t_{H}$ being the history horizon.

The drag coefficient $C_{D}$ is usually determined as a function of the particle Reynolds number

(13) Re=ρfdpvμf.

For instance, in the range of intermediate particle Reynolds numbers (0.1<Re<103), the following relation due to Schiller and Naumann [15] is commonly used:

(14) CDSN=24Re(1+0.15Re0.687).

Observe that in the limit of extremely small Reynolds numbers (Re≪1), formula (14) reduces to Stokes' law

(15) CD≃24Re,

so that, in view of (13) and (15), formula (9) produces Stokes' drag

$$F_{D}≃3\pi \mu_{f}d_{p}v.$$

It is also to note that in the range of very low Reynolds numbers (Re<0.1), there known other approximations for the drag coefficient, which are more accurate than (14) and (15).

Empirical relationships for the drag coefficient $C_{D}$ as a function of Reynolds number Re are determined based on steady-state flows. However, it should be emphasized that, besides the steady drag force $F_{D}$, the dynamic balance equation (5) contains the Basset history force $F_{H}$ and the added mass force $F_{\mathrm{AM}}$, both of which depend on the instantaneous velocity values of a settling particle and, thereby, reflect transient effects in the sedimentation.

### 2.2 Steady state and terminal velocity

In the regime of steady-state motion, when v=const, the differential equation (7), in view of Eqs. (8)–(10), simplifies as

(16) $$\frac{4}{3}({\overset{¯}{\rho }}_{p}-\rho_{f})d_{p}g=\rho_{f}v^{2}C_{D}(v),$$

where, according to Eqs. (13) and (14) for the Schiller–Naumann model (SN), we have

(17) $$C_{D}^{\mathrm{SN}}(v)=\frac{24\mu_{f}}{\rho_{f}d_{p}v}\{1+0.15{\left(\frac{\rho_{f}d_{p}v}{\mu_{f}}\right)}^{0.687}\}.$$

Equations (16) and (17) determine the so-called terminal (or steady-state) velocity, $v_{s}$.

On the other hand, provided the terminal velocity is measured in experiment, Eqs. (16) and (17) serve as a basis for determining the mean particle density ${\overset{¯}{\rho }}_{p}$.

In view of (15), the Stokes terminal velocity is given by

(18) $$v_{\mathrm{Stokes}}=\frac{g}{18\mu_{f}}({\overset{¯}{\rho }}_{p}-\rho_{f})d_{p}^{2}.$$

Based on the Stokes terminal velocity $v_{\mathrm{Stokes}}$ and the actual steady-state velocity $v_{s}$, we introduce the following specific Reynolds numbers:

(19) ReSt=ρfdpμfvStokes,Res=ρfdpμfvs.

It is to note that, while the Stokes terminal velocity $v_{\mathrm{Stokes}}$ is defined by formula (18), the actual terminal velocity $v_{s}$ is determined by Eqs. (16) and (17).

### 2.3 Drag coefficient correction factor

In light of Stokes' law (15), we put

(20) cD(Re)=Re24CD(Re).

For the Schiller–Naumann (SN) approximation of the drag coefficient (14), the drag coefficient correction factor is given by formula (20).

Based on thorough analysis of experimental data, it is generally accepted [16] that the Clift–Grace–Weber (CGW) correlations of drag coefficient with Reynolds number given in [11] are ones of the best available approximations CD(Re) of the standard drag curve. In particular, we have

(21) cDCGW(Re)={1+1128Re,Re≤0.01,1+0.131Re0.82−0.05log10⁡(Re),0.01<Re≤20,1+0.193Re0.6305,20<Re≤260.

At the same time, Brown and Lawler [16] suggested their own single-formula approximation for CD(Re) in the range Re<2⋅105, from where it follows that

(22) cDBL(Re)=1+0.150Re0.681+0.40Re224(Re+8710).

In their review of 24 equations for of the standard drag curve. In particular, it was found that [17] the single-formula approximation for the drag coefficient of a sphere developed by Flemmer and Banks [18] is most closely matches the CGW correlations (21) in the range Re<3⋅105. The Flemmer–Banks approximation for the drag coefficient correction factor has the following form:

(23) cDFB(Re)=10E,E=0.261Re0.369−0.105Re0.431−0.1241+(log10⁡Re)2.

Based on the analysis of experimental data on sedimentation of small spherical particles, the following relationship for calculating the drag coefficient of a spherical particle has been presented [19]:

(24) CDTHG(Re)=A1+A2Re+A3Re2+A4Re0.1+A5Re0.2.

Here, $A_{1}=2.689$, $A_{2}=21.683$, $A_{3}=0.131$, $A_{4}=-10.616$, and $A_{5}=12.216$.

**Figure 1:**
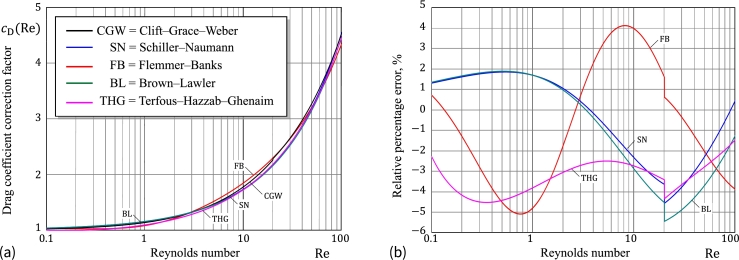
(a) Drag coefficient correction factor vs. Reynolds number; (b) Relative percentage errors of *c*_D_(Re) with respect to the Clift–Grace–Weber correlations cDCGW(Re) for the Schiller–Naumann (29), Brown–Lawler (22), and Flemmer–Banks approximations (23).

Fig. 1a shows the variations of cDCGW(Re), cDSN(Re), and cDFB(Re) in the range of interest for Reynolds number (0.1≤Re≤100). Fig. 1b presents the relative percentage errors of the approximations cDSN(Re) and cDFB(Re) with respect to cDCGW(Re). For what follows, it is important to observe that the drag coefficient correction factor cD(Re) is an *increasing* function of Reynolds number Re.

**Figure 2:**
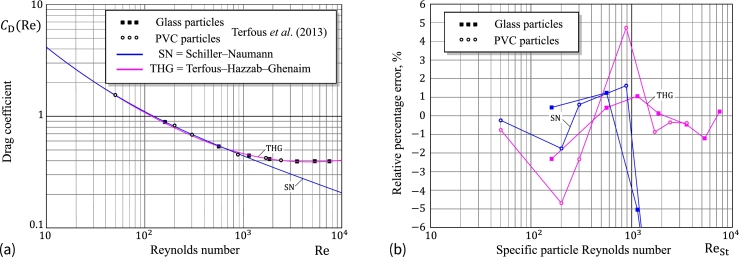
(a) Drag coefficient vs. Reynolds number; (b) Relative percentage errors of *C*_D_(Re) with respect to the experimental results obtained by Terfous et al. [19] for the Schiller–Naumann (14) and the Terfous–Hazzab–Ghenaim (24) approximations.

It goes without saying that the empirical correlations considered here do not exhaust all of the approximate formulas for drag coefficient available in the literature (see a recent extended review [20]). While their accuracy has been tested against the standard drag curve via the Clift–Grace–Weber correlations, it is still of practical interest to compare predictions of the Schiller–Naumann (SN) approximation with experimental results obtained by Terfous et al. [19] for small spherical particles ranging in size from 1 to 10 millimeters (see Fig. 2a).

As it is seen from Fig. 2b, an accuracy within 5 per cent can be expected from the Schiller–Naumann approximation in the range of intermediate particle Reynolds numbers (0.1<Re<103). Observe that in the shorter range of interest (0.1<Re<800), the SN formula possesses the uncertainty of only a couple of percents. Finally, it should be emphasized [6] that the Schiller–Naumann approximation is recommended for the use in the range Re<800.

### 2.4 Governing integro-differential equation

Following [21], we nondimensionalize Eq. (7) by using the terminal Stokes velocity $v_{\mathrm{Stokes}}$, defined by Eq. (18) and the so-called particle characteristic relaxation time, $t_{r}$, defined as follows:

(25) $$t_{r}=\frac{m_{p}}{3\pi \mu_{f}d_{p}}.$$

In this way, we introduce non-dimensional variables

(26) $$\upsilon =\frac{v}{v_{\mathrm{Stokes}}},\;\tau =\frac{t}{t_{r}}.$$

By collecting formulas (7), (9), (11), and (12), in view of Eqs. (26), we arrive at the governing integro-differential equation in the nondimensional form

(27) (1+χ2)dυdτ=1−υcD(υReSt)−9χ2π∫τ−τwindowτdυ(τ′)dτ′dτ′τ−τ′,

where we have introduced the notation

(28) $$\chi =\frac{\rho_{f}}{{\overset{¯}{\rho }}_{p}},$$

the Schiller–Naumann drag correction factor (see Eq. (14)) is

(29) cDSN(Re)=1+0.15Re0.687,

and the lower limit of integration is given by

τ−τwindow=min⁡{0,τ−τH},τH=18χ(0.502υReSt+0.123)2.

The initial condition for the nonlinear integro-differential equation (27) is taken to be

(30) υ(0)=0.

The Picard method based numerical algorithm for solving the initial problem (27), (30) was suggested by Sobral et al. [21]. In their paper, both linear and nonlinear equations without memory effects have been considered and the separate effect of the Basset history forces has been studied in detail.

### 2.5 Relative terminal velocity

It can be shown that $\upsilon (\tau )\to \upsilon_{s}$ as τ→∞, where $\upsilon_{s}$ is the dimensionless steady-state velocity, which, according to Eqs. (16), (17), and ${\text{(26)}}_{1}$, solves the equation

(31) υscD(υsReSt)=1,

where ReSt is defined by Eq. ${\text{(19)}}_{1}$, and cD(Re) is given by Eq. (29).

**Figure 3:**
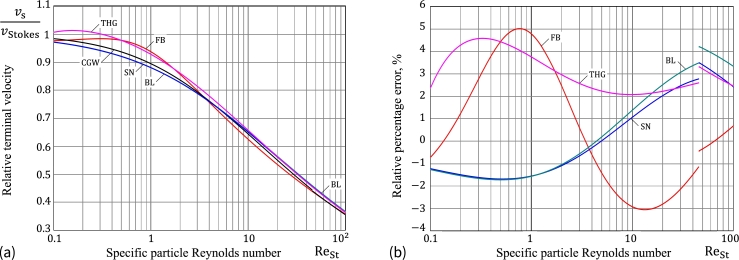
(a) Relative terminal velocity vs. specific particle Reynolds number; (b) Relative percentage errors of *υ*_s_(Re_St_) with respect to the solution υsCGW(ReSt) according to the Clift–Grace–Weber correlations cDCGW(Re) for the Schiller–Naumann (29), Brown–Lawler (22), and Flemmer–Banks (21) approximations of the drag coefficient correction factor.

Fig. 3a shows the variations of υsCGW(ReSt), υsSN(ReSt), and υsFB(ReSt) in the range 0.1≤ReSt≤100 for the specific particle Reynolds number ReSt. It is of paramount importance to note that the Flemmer–Banks approximation (21) for the correction drag coefficient factor predicts a non-monotonic variation of $\upsilon_{s}$ as a function of ReSt in the range 0.1≤ReSt≤1, which is an artifact of the FM approximation, apparently resulting from fitting the standard drag curve with a single-formula approximation in a very wide range Re<3⋅105.

Fig. 3b presents the relative percentage errors of the approximations υsSN(ReSt) and υsFB(ReSt) with respect to υsCGW(ReSt).

### 2.6 Settling time and distance

The dimensionless settling time, $\tau_{n\text{\%}}$, is defined as the dimensionless time moment $\tau =\tau_{n\text{\%}}$ after which the relative percentage difference $(\upsilon_{s}-\upsilon (\tau ))/\upsilon_{s}\times 100\text{\%}$ falls below a certain threshold of *n*% (e.g., 5% or less depending on the assumed level of accuracy). Accordingly, in view of ${\text{(26)}}_{2}$, the dimensional settling time of sedimentation will be given by

(32) $$t_{n\text{\%}}=t_{r}\tau_{n\text{\%}},$$

where $t_{r}$ is the characteristic relaxation time defined by Eq. (25).

The corresponding settling distance is introduced by the formula

(33) $$l_{n\text{\%}}=\int_{0}^{t_{n\text{\%}}}v(t)\;dt$$

as the distance traveled by the particle when it approaches the steady state.

In view of (26), Eq. (33) can be rewritten in the form

(34) $$l_{n\text{\%}}=v_{\mathrm{Stokes}}t_{r}\int_{0}^{\tau_{n\text{\%}}}\upsilon (\tau )\;d\tau .$$

In regard to the method of sedimentation, it is of paramount importance to derive upper estimates for the settling parameters $t_{n\text{\%}}$ and $l_{n\text{\%}}$ in order to assure that the terminal velocity os experimentally measured when a sedimenting particle practically — that is, within the assumed accuracy — reached the steady-state motion.

With this aim, we consider the following linear integro-differential equation

(35) $$A\frac{dV}{d\tau }=1-CV-B\int_{0}^{\tau }\frac{dV(\tau^{\prime })}{d\tau^{\prime }}\frac{d\tau^{\prime }}{\sqrt{\tau -\tau^{\prime }}},$$

where we have introduced the notation

(36) A=1+χ2,B=9χ2π,C=cD(υsReSt).

Based on Tchaplygin's theorem on differential inequalities [22], it can be shown that V(τ)<υ(τ) for any τ>0, provided that both functions V(τ) and υ(τ) satisfy the same initial condition (30). Moreover, by the definition of the coefficient C, we have that $V(\tau )\to \upsilon_{s}$ as τ→∞, so that both functions V(τ) and υ(τ) approach the same steady state. However, the so-called relaxation time for V(τ) to reach $\upsilon_{s}$ will be longer than that for υ(τ).

We would like to emphasize that, taking into account Eq. (31), we can write

(37) $$C=\frac{1}{\upsilon_{s}},$$

and thus, in view of (37), Eq. (35) remains absolutely the same regardless of the drag coefficient model.

We solve the initial problem (35), (30) using Laplace transform technique, and, in view of Eq. (31), arrive at the following representation of the Laplace transform, $\overset{˜}{\upsilon }(s)$, of the dimensionless particle velocity:

(38) $$\overset{˜}{\upsilon }(s)=\frac{1}{s[As+B\sqrt{\pi s}+1/\upsilon_{s}]}.$$

Here, *s* is the Laplace transform variable.

Let us introduce the notation

(39) $$s_{1,2}=\frac{1}{2A}(-\sqrt{\pi }B\pm i\sqrt{\frac{4A}{\upsilon_{s}}-\pi B^{2}}),$$

where *i* is the imaginary unit.

Now, utilizing inverse Laplace tables [23], we invert Eq. (38) to find the approximate dimensionless variable velocity of the particle in the form

(40) $$V(\tau )=\frac{1}{A(s_{2}-s_{1})}\{\frac{1}{s_{1}}-\frac{1}{s_{2}}+\frac{1}{s_{2}}\exp(s_{2}^{2}\tau )\mathrm{erfc}(-s_{2}\sqrt{\tau })-\frac{1}{s_{1}}\exp(s_{1}^{2}\tau )\mathrm{erfc}(-s_{1}\sqrt{\tau })\},$$

where the complex parameters $s_{1}$ and $s_{2}$ are given by (39), and erfc(z) is the complementary error function, that is

$$\mathrm{erfc}(z)=\frac{2}{\sqrt{\pi }}\int_{z}^{\infty }e^{-\zeta^{2}}d\zeta =1-\mathrm{erf}(z),\;\mathrm{erf}(z)=\frac{2}{\sqrt{\pi }}\int_{0}^{z}e^{-\zeta^{2}}d\zeta .$$

We note that the solution given by formula (40) can be expressed in terms of the Faddeeva function $w(z)=\exp(-z^{2})\mathrm{erfc}(-iz)$ [24], [25]. In this way, using the asymptotic representation $w(z)∼i/(\sqrt{\pi }z)$ for large |z|, we establish the simple asymptotic formula

(41) $$V(\tau )∼\frac{1}{A|s_{1}{|}^{2}}(1+\frac{2ℜ(s_{1})}{\sqrt{\pi \tau }}),\;\tau \to \infty ,$$

where $ℜ(s_{1})$ and $\left|s_{1}\right|$ are the real part and the modulus of the complex number $s_{1}$, respectively.

In view of (36) and (39), formula (41) can be simplified as

(42) $$V(\tau )∼\upsilon_{s}(1-\sqrt{\frac{9\chi }{2\pi }}\frac{\upsilon_{s}}{\sqrt{\tau }}),\;\tau \to \infty ,$$

where *χ* is the density ratio defined by (28).

**Figure 4:**
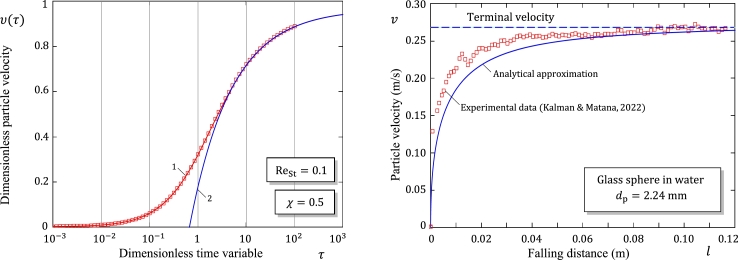
(a) Time evolution of the dimensionless particle velocity for the nonlinear problem with memory effects: Comparison of the numerical solution obtained by Sobral et al. [21] (square symbols) with the analytical solutions given by formulas (40) (Curve 1) and (42) (Curve 2). (b) Comparison of the analytical approximate solution based on formula (40) with the experimental data (square symbols) presented by Kalman and Matana [26].

Fig. 4a illustrates the accuracy of the asymptotic approximation (42) for the solution of the nonlinear integro-differential equation (27) without window (t.e., when the lower limit of integration in the history integral equals zero) in the case of Oseen approximation for the drag coefficient CD(Re)=(24/Re)(1+3Re/16), which is generally recommended for Re≤1, but has a good matching with the experimental data of spheres up to Re=10 [6].

Fig. 4b illustrates the variation of the particle velocity as a function of distance in a sample experimental measurement performed by Kalman and Matana [26] for a glass particle ($\rho_{p}=2495\;\mathrm{kg}\;m^{-3}$) falling in water ($\rho_{f}=986\;\mathrm{kg}\;m^{-3}$, $\mu_{f}=0.0012\;\mathrm{Pa}\cdot s$, and $g=9.81\;m\;s^{-2}$). It should be noted that though the experimental results [26] are obtained for glass particles whose density is markedly different from that of the fluid, the terminal Reynolds number is evaluated to be about 500, which falls well within the range of applicability of the developed mathematical model. As it is expected, the developed approximate solution underestimates the particle velocity, however, the discrepancy diminishes as the transient motion approaches a steady state.

### 2.7 A priori estimates for the settling time and distance

So, let $t_{n\text{\%}}$ denote the time moment such that

$$v(t_{n\text{\%}})=(1-\frac{n\text{\%}}{100\text{\%}})v_{s}.$$

By making use of the asymptotic formula (42), we readily find that

(43) $$\sqrt{\tau_{n\text{\%}}}≃\frac{300\sqrt{\chi }}{\sqrt{2\pi }n}\upsilon_{s},$$

and thus, in view of (25), (28), and (32), we obtain the upper estimate

(44) $$t_{n\text{\%}}≃\frac{2500}{\pi n^{2}}\frac{\rho_{f}d_{p}^{2}}{\mu_{f}}\upsilon_{s}^{2}.$$

In the special case, for n%=1%, since 2500/π≈796, in light of the inequality $\upsilon_{s}<1$, the approximate formula (44) fairly agrees with the conservative upper estimate given by formula (4).

**Figure 5:**
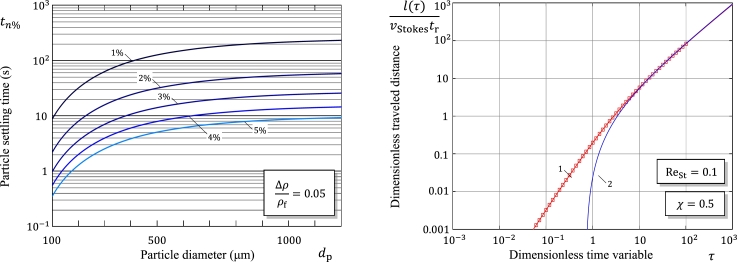
(a) Settling time for spherical particles in water versus particle diameter. (b) Time evolution of the particle traveled distance for the nonlinear problem with memory effects: Comparison of the numerical solution obtained from the solution presented by Sobral et al. [21] (square symbols) with the analytical solutions based on formulas (45), (40) (Curve 1) and (42) (Curve 2).

Fig. 5a illustrates the application of formula (44) in the case of sedimentation in water ($\rho_{f}=0.997\;g/{\mathrm{cm}}^{3}$ and $\mu_{f}=0.89\cdot 10^{-3}\;\mathrm{Pa}\cdot s$.

Further, in view of (26), (33), and (34), the relative settling distance can be represented in the form

(45) $$\frac{l_{n\text{\%}}}{v_{\mathrm{Stokes}}t_{r}}=\int_{0}^{\tau_{n\text{\%}}}\upsilon (\tau )\;d\tau .$$

Again, by using the asymptotic formula (42) for estimating the relative velocity υ(τ), we obtain

(46) $$l_{n\text{\%}}\approx v_{\mathrm{Stokes}}t_{r}\upsilon_{s}{\left(\sqrt{\tau_{n\text{\%}}}-\sqrt{\tau_{0}}\;\right)}^{2},$$

where we have introduced the notation

(47) $$\sqrt{\tau_{0}}=\frac{3\sqrt{\chi }}{\sqrt{2\pi }}\upsilon_{s}.$$

Fig. 5b illustrates the accuracy of the asymptotic approximation (47) for the solution (45) of the nonlinear integro-differential equation (27) without window in the case of Oseen approximation for the drag coefficient.

Thus, taking into account that $\sqrt{\tau_{0}}/\sqrt{\tau_{n\text{\%}}}=n/100$ (see relations (43) and (47)) and utilizing formulas (19), (25), (28), and (43), we can represent formula (46) in the form

(48) ln%≈2500πn2(1−n100)2ReStυsdp.

In particular, for n%=1%, the above formula yields l1%≈780ReStυsdp.

It is to note that the relative settling velocity $\upsilon_{s}$, which is defined as the root of Eq. (31), depends on the Reynolds number ReSt evaluated for the Stokes velocity.

**Figure 6:**
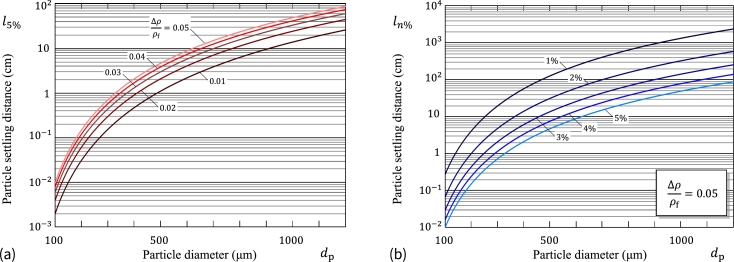
(a) Particle settling distance with 5% accuracy vs. particle diameter for different values of the relative density difference; (b) Particle settling distance vs. particle diameter for the fixed relative density difference.

The application of formula (48) in the case of gravimetric sedimentation in water for particles with relatively small values of the difference Δ*ρ* between the particle density ${\overset{¯}{\rho }}_{p}$ and the density of the fluid $\rho_{f}$ is illustrated in Fig. 6a (for $l_{5\text{\%}}$) and Fig. 6b (for $l_{1\text{\%}},l_{2\text{\%}},\ldots ,l_{5\text{\%}}$).

## 3 Discussion

So, when attempting to apply the gravimetric sedimentation method for determining the mean density of starch microparticles, first it is necessary to evaluate the specific particle Reynolds number ReSt, which, in view of (18) and (19), is given by

(49) ReSt=ρf2g18μf2(ρ¯fρf−1)dp3.

Observe that three factors can be distinguished on the right-hand side of Eq. (49). Namely, the first factor $\rho_{f}^{2}g/(18\mu_{f}^{2})$ is fully determined by the fluid properties, the second factor represents the relative density difference $\Delta \rho /\rho_{f}$, and the third factor $d_{p}^{3}$ is proportional to the particle volume. In the case of starch microparticles, when the particle density varies in a rather limited range, the particle size becomes the most decisive factor in determining the regime of relative fluid flow around the sedimenting particle.

While native potato starch granules are not spherical (see, e.g., Fig. 1 in [27]), isolated starch microspheres at the excess of water are almost perfectly spherical (see Fig. 4 in [8]). Still possible deviations from the ideal spherical shape of a particle used in the sedimentation experiments can be taken into account, using the concept of particle sphericity [28]. It is known that as the deviation from sphericity of a falling particle increases, the drag coefficient increases (for the same Reynolds number), and thus, the settling time decreases. The latter means that the conservative estimates obtained above still can be applied to estimate the settling time.

**Figure 7:**
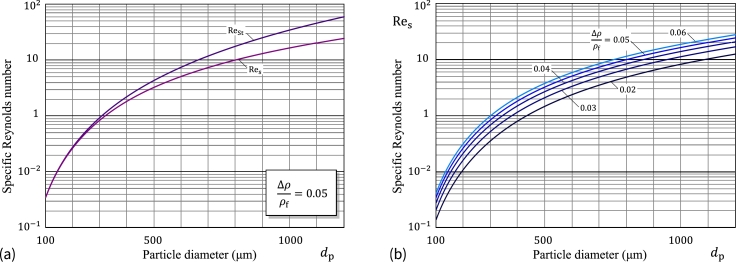
(a) Specific Reynolds numbers Re_St_ and Re_s_ (based on the Clift–Grace–Weber correlations) for starch microspheres in water as a function of the sphere diameter *d*_p_; (b) Specific particle Reynolds number corresponding to the terminal velocity as a function of the sphere diameter.

Fig. 7a illustrates the behavior of the specific Reynolds numbers ReSt and Res for starch microspheres in water as a function of the sphere diameter $d_{p}$ based on the CGW model.

Fig. 7b shows the variation of the specific particle Reynolds number Res versus the particle diameter $d_{p}$ for a number of characteristic values of the density ratio. It is readily seen that in the chosen range of the particle sizes, the values of ReSt fall in both the so-called [29] laminar (Re<0.2) and transitional (0.2<Re<750) regions, and thus the relative terminal velocity $\upsilon_{s}$ should be determined by solving Eq. (31) with an approximate choice of the drag correction factor cD(Re).

Whereas the analytical correlations of drag coefficient CD(Re) formulated in [11] are widely regarded to be one of the best approximations of the standard drag curve, it is shown that in the limited range 0.1<Re<100, the models suggested by Schiller and Naumann [15], and Brown and Lawler [16] also yield reliable results with a few percent error. However, it should be emphasized that the approximation suggested by Flemmer and Banks [18] is not suitable for applications for ReSt≤3.

**Figure 8:**
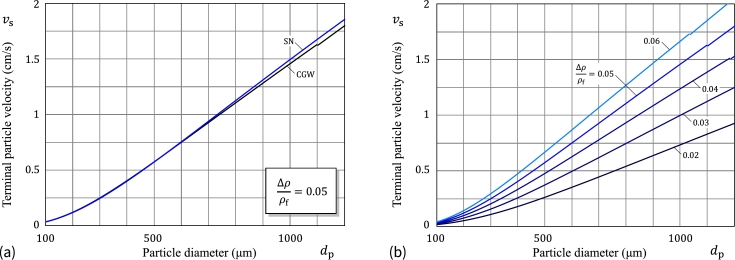
(a) Terminal (settling) velocity *v*_s_ for starch microspheres in water as a function of the sphere diameter *d*_p_: Predictions are based on the Clift–Grace–Weber (CGW) correlations and the Schiller–Naumann (SN) formula; (b) Terminal velocity for starch microspheres in water as a function of the sphere diameter (based on the CGW correlations).

Fig. 8a shows the theoretical predictions for the terminal velocity based on the CGW and SN models. We note that the latter model underestimates the values of $v_{s}$ (for $d_{d}$ less than 500μm, with the maximum relative absolute percentage error 1.7%) and overestimates the values of $v_{s}$ (for $d_{d}$ greater than 550 μm, with the maximum relative percentage error 3.5%). The terminal velocity for starch microspheres in water as a function of the sphere diameter based on the CGW model is presented in Fig. 8b.

It is of interest to observe that the settling time $t_{n\text{\%}}$ (see formula (44)) does not depend neither on the particle density ${\overset{¯}{\rho }}_{p}$ nor on the gravitational acceleration *g*. The latter means that the duration of the process of accelerating the particle to its terminal velocity is fully governed by the viscous properties of the fluid medium.

Another important consequence of formula (44) is that an insignificant lowering of accuracy from 1% to 2% reduces the required settling time from $t_{1\text{\%}}$ to $t_{2\text{\%}}$ by four times, since $t_{1\text{\%}}/t_{n\text{\%}}=n^{2}$. And about the same statement can be made for settling distance $l_{n\text{\%}}$ given by formula (48).

**Figure 9:**
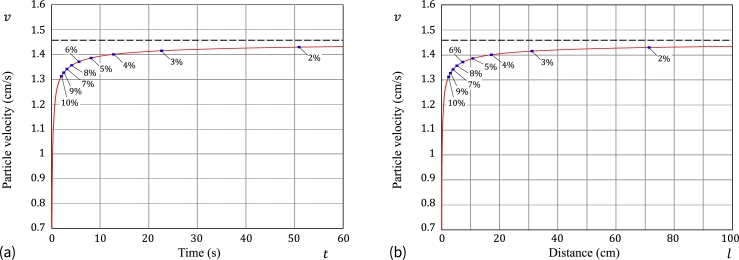
Theoretical predictions for the particle settling velocity *v* in water for a starch microsphere of diameter *d*_p_ = 1mm and relative density difference ratio Δ*ρ*/*ρ*_f_ = 0.05 as a function (a) of time and (b) settling distance, which are based on the approximate model (40) and the CGW correlations (21).

Fig. 9 illustrates the non-stationary movement of a sedimenting particle ($d_{p}=1\mathrm{mm}$ and $\Delta \rho /\rho_{f}=0.05$) as it approaches the steady state via monitoring the starch particle settling velocity *v* as a function of time (see Fig. 9a) and settling distance (see Fig. 9b). It is of interest to observe that in order to increase the accuracy from 3% to 2%, it will be necessary to roughly double the sedimenting distance.

**Figure 10:**
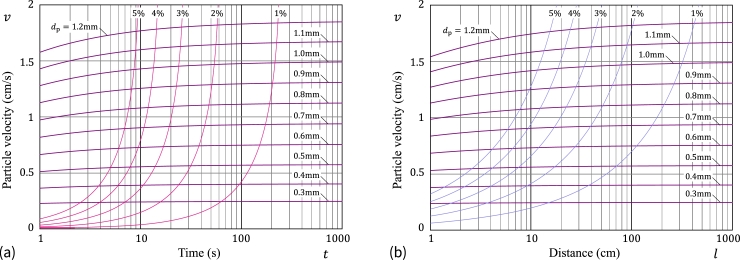
Theoretical predictions for the particle settling velocity *v* in water for a starch microsphere of different diameter and fixed relative density difference ratio Δ*ρ*/*ρ*_f_ = 0.05 as a function (a) of time and (b) settling distance, which are based on the asymptotic solution (42) and the SN approximation (14).

The variation of the particle settling velocity *v* in water for a starch microsphere with the fixed ratio $\Delta \rho /\rho_{f}=0.05$ and different diameters is presented in Fig. 10a (*v* versus *t*) and Fig. 10b (*v* versus *l*).

## 4 Conclusion

To conclude, the developed theoretical framework is aimed at accurate determining the mean density of a starch microsphere suing the method of gravimetric sedimentation. The specificity of starch microparticles determines the range of specific particle Reynolds numbers involved in the analysis. At the same time, the general mathematical scheme of analysis is applicable for microspheres made of other materials, provided the underlying assumptions are valid. A main novelty of this study consists in explicit *a priori* estimates for the settling time and distance of the non-steady stage of sedimentation.

## CRediT authorship contribution statement

Ivan Argatov: Performed the experiments; Analyzed and interpreted the data; Wrote the paper.

Nedim Krcic: Conceived and designed the experiments; Contributed reagents, materials, analysis tools or data.

Vitaly Kocherbitov: Analyzed and interpreted the data; Wrote the paper.

## Declaration of Competing Interest

The authors declare that they have no known competing financial interests or personal relationships that could have appeared to influence the work reported in this paper.

## Data Availability

Data will be made available on request.

## References

[1] Rodrigues A., Emeje M. (2012). Recent applications of starch derivatives in nanodrug delivery. Carbohydr. Polym..

[2] Wojtasz J., Carlstedt J., Fyhr P., Kocherbitov V. (2016). Hydration and swelling of amorphous cross-linked starch microspheres. Carbohydr. Polym..

[3] Wacholder E. (1973). Sedimentation in a dilute emulsion. Chem. Eng. Sci..

[4] Ma J., Wu Y., Liu Y., Ji Y., Yang M., Zhu H. (2021). Cell-sorting centrifugal microfluidic chip with a flow rectifier. Lab Chip.

[5] Merkus H.G. (2009). Particle Size Measurements: Fundamentals, Practice, Quality.

[6] Dey S., Ali S.Z., Padhi E. (2019). Terminal fall velocity: the legacy of Stokes from the perspective of fluvial hydraulics. Proc. R. Soc. A.

[7] Mann H., Mueller P., Hagemeier T., Roloff C., Thévenin D., Tomas J. (2015). Analytical description of the unsteady settling of spherical particles in Stokes and Newton regimes. Granul. Matter.

[8] Digaitis R., Falkman P., Oltner V., Briggner L., Kocherbitov V. (2022). Hydration and dehydration induced changes in porosity of starch microspheres. Carbohydr. Polym..

[9] M. Cretella, M. Fazilati, N. Krcic, I. Argatov, V. Kocherbitov, Determination of density of hydrated starch microspheres from sedimentation experiments using non-Stokes drag coefficient, Starch, submitted for publication. 10.3390/gels10040277 PMC11049465 38667696

[10] Atyabi F., Manoochehri S., Moghadam S.H., Dinarvand R. (2006). Cross-linked starch microspheres: effect of cross-linking condition on the microsphere characteristics. Arch. Pharm. Res..

[11] Clift R., Grace J.R., Weber M.E. (1978).

[12] Bernhardt C. (2006).

[13] Loth E., Dorgan A.J. (2009). An equation of motion for particles of finite Reynolds number and size. Environ. Fluid Mech..

[14] Dorgan A.J., Loth E. (2007). Efficient calculation of the history force at finite Reynolds numbers. Int. J. Multiph. Flow.

[15] Schiller L., Naumann A. (1935). A drag coefficient correlation. Z. Ver. Dtsch. Ing..

[16] Brown P.P., Lawler D.F. (2003). Sphere drag and settling velocity revisited. J. Environ. Eng..

[17] Zhang L., Honaker R., Liu W., Men D., Chen J. (2015). Calculation of terminal velocity in transitional flow for spherical particle. Int. J. Min. Sci. Technol..

[18] Flemmer R.L.C., Banks C.L. (1986). On the drag coefficient of a sphere. Powder Technol..

[19] Terfous A., Hazzab A., Ghenaim A. (2013). Predicting the drag coefficient and settling velocity of spherical particles. Powder Technol..

[20] Goossens W.R.A. (2019). Review of the empirical correlations for the drag coefficient of rigid spheres. Powder Technol..

[21] Sobral Y.D., Oliveira T.F., Cunha F.R. (2007). On the unsteady forces during the motion of a sedimenting particle. Powder Technol..

[22] Wilkins J.E. (1947). The converse of a theorem of Tchaplygin on differential inequalities. Bull. Am. Math. Soc..

[23] Bateman H., Erdélyi A. (1953).

[24] Gautschi W. (1970). Efficient computation of the complex error function. SIAM J. Numer. Anal..

[25] Weideman J.A.C. (1994). Computation of the complex error function. SIAM J. Numer. Anal..

[26] Kalman H., Matana E. (2022). Terminal velocity and drag coefficient for spherical particles. Powder Technol..

[27] Carlstedt J., Wojtasz J., Fyhr P., Kocherbitov V. (2015). Understanding starch gelatinization: the phase diagram approach. Carbohydr. Polym..

[28] Haider A., Levenspiel O. (1989). Drag coefficient and terminal velocity of spherical and nonspherical particles. Powder Technol..

[29] Majumder A.K. (2007). Settling velocities of particulate systems—a critical review of some useful models. Min. Metall. Explor..

